# Promotion of Vascular Morphogenesis of Endothelial Cells Co-Cultured with Human Adipose-Derived Mesenchymal Stem Cells Using Polycaprolactone/Gelatin Nanofibrous Scaffolds

**DOI:** 10.3390/nano8020117

**Published:** 2018-02-18

**Authors:** Yun-Min Kook, Hyerim Kim, Sujin Kim, Kangwon Lee, Chan Yeong Heo, Min Hee Park, Won-Gun Koh

**Affiliations:** 1 Department of Chemical and Biomolecular Engineering, Yonsei University, 50 Yonsei-ro, Seodaemun-gu, Seoul 120-749, Korea; yunmin@yonsei.ac.kr; 2 Program in Nanoscience and Technology, Graduate School of Convergence Science and Technology, Seoul National University, Seoul 08826, Korea; hyerimkin@snu.ac.kr (H.K.); sujin.k@snu.ac.kr (S.K.); minheepark@snu.ac.kr (M.H.P.); 3 Advanced Institutes of Convergence Technology, Gyeonggi-do 16229, Korea; 4 Department of Plastic and Reconstructive Surgery, Seoul National University College of Medicine, Seoul 03080, Korea; lionheo@snu.ac.kr; 5 Department of Plastic and Reconstructive Surgery, Seoul National University Bundang Hospital, Seongman 13620, Korea

**Keywords:** co-culture, endothelial cells, mesenchymal stem cells, electrospun nanofiber, angiogenesis

## Abstract

New blood vessel formation is essential for tissue regeneration to deliver oxygen and nutrients and to maintain tissue metabolism. In the field of tissue engineering, in vitro fabrication of new artificial vessels has been a longstanding challenge. Here we developed a technique to reconstruct a microvascular system using a polycaprolactone (PCL)/gelatin nanofibrous structure and a co-culture system. Using a simple electrospinning process, we fabricated three-dimensional mesh scaffolds to support the sprouting of human umbilical vein endothelial cells (HUVECs) along the electrospun nanofiber. The co-culture with adipose-derived mesenchymal stem cells (ADSCs) supported greater sprouting of endothelial cells (ECs). In a two-dimensional culture system, angiogenic cell assembly produced more effective direct intercellular interactions and paracrine signaling from ADSCs to assist in the vascular formation of ECs, compared to the influence of growth factor. Although vascular endothelial growth factor and sphingosine-1-phosphate were present during the culture period, the presence of ADSCs was the most important factor for the construction of a cell-assembled structure in the two-dimensional culture system. On the contrary, HUVECs co-cultured on PCL/gelatin nanofiber scaffolds produced mature and functional microvessel and luminal structures with a greater expression of vascular markers, including platelet endothelial cell adhesion molecule-1 and podocalyxin. Furthermore, both angiogenic factors and cellular interactions with ADSCs through direct contact and paracrine molecules contributed to the formation of enhanced engineered blood vessel structures. It is expected that the co-culture system of HUVECs and ADSCs on bioengineered PCL/gelatin nanofibrous scaffolds will promote robust and functional microvessel structures and will be valuable for the regeneration of tissue with restored blood vessels.

## 1. Introduction

New blood vessel formation is crucial to supplying nutrients and oxygen to cells and to remove waste products during the engineering of new tissues. For the successful development of a functional vascular network, adequate and clinically applicable biomaterials are required to provide a suitable environment for the construction of new blood vessels and to avoid cellular toxicity. Strategies that have been used to enhance vascularization include the delivery of angiogenic factors such as vascular endothelial growth factor (VEGF) and sphingosine-1-phosphate (S1P) into biomaterials as therapeutic agents to promote blood vessel formation [1,2,3,4].

Therapeutic approaches for microvasculature formation using mesenchymal stem cells (MSCs) have been developed. MSCs have the potential to differentiate into multiple lineages including vascular and endothelial lineages [5,6,7]. Additionally, MSCs exhibit the features and characteristics of the perivascular cells known as pericyte [8]. Pericytes contribute to the stabilization, maintenance, and hemodynamic processes of blood vessels by acting as angiogenic stimuli and guiding the sprouting of vessels [9]. MSCs may similarly contribute to vascular formation and could be an alternative cell source in vascular grafts or tissue engineering. More recently, cell-based approaches proposed to exploit the intrinsic vasculogenic or angiogenic capacity of endothelial cells (ECs) or endothelial progenitor cells with intercellular interactions with MSCs [10,11,12,13,14,15]. The collective data indicate the capability of co-cultures with MSCs and ECs in the development of blood vessels in vivo after cell transplantation. Both cell types are appealing sources for generating functional vascular networks for tissue regeneration applications [16]. One of the MSCs, adipose-derived mesenchymal stem cells (ADSCs) have emerged as a novel source of neovascularization that secretes various angiogenic factors (including VEGF, angiopoietin, and a variety of microRNAs) more than bone-marrow-derived MSCs [17,18]. This finding suggests that ADSCs may be a powerful candidate for blood vessel regeneration.

The design of cellular scaffolds using synthetic and natural polymers and extracellular matrix (ECM) components is a crucial factor for the production of functionally-engineered microvessel structures in vitro. These polymers and the ECM can provide appropriate cell adhesion sites and promote cell proliferation and protein synthesis during vasculogenesis and angiogenesis. In the most current models of engineered blood vessel development, cells are cultured in or on biomaterial-based porous scaffolds such as matrigel, hydroxyapatite, calcium phosphate, beta-tricalcium phosphate (β-TCP), and collagen; all these are favorable microenvironments for vascular morphogenesis [19,20]. These various approaches to promoting vascular formation have been successful in the production of microvessels. However, these scaffolds have some properties that are not ideal for tissue engineering applications and which have precluded them from clinical trial evaluation; these include poor mechanical support of cells, immunogenic problems, and inadequate mimicry of the ECM environment. 

These barriers might be overcome by the blending of the ECM component protein, and a Food and Drug Administration (FDA) approved polymer to make functional cellular scaffolds. A recent approach for the design of tissue engineering scaffolds is the electrospun fiber scaffold. This method improves cell adhesion, proliferation, and stem cell differentiation. Electrospinning successfully provides three-dimensional (3D) culture conditions and suitable mechanical durability that mimics the fibrous hierarchical structure of the ECM [21,22,23,24,25,26]. For several potential tissue engineering applications and clinical trials, it is desirable to develop scaffolds that are biocompatible and mechanically stable, which can control the scale from the microscopic to nanoscopic level to mimic the real tissue microenvironments. 

To accomplish this, we fabricated electrospun nanofibers consisting of blended polycaprolactone (PCL) and gelatin as permissive scaffolds for the formation of functional vascular networks. Additionally, co-culturing of human umbilical vein endothelial cells (HUVECs) and human adipose-derived mesenchymal stem cells (hADSCs) was performed to explore the efficacy of the intercellular interactions in enhancing blood vessel formation through direct cell–cell contact and paracrine signaling on the nanofibrous scaffolds. VEGF and S1P were used as angiogenic factors in the cell culture medium, as the biochemical cues to promote the vascular morphogenesis of HUVECs. After construction of the cellular PCL/gelatin nanofiber scaffolds, we investigated the vasculogenic or angiogenic activities of the monocultured or co-cultured HUVECs, such as cell proliferation, morphogenesis, protein expression, and lumen formation. The results demonstrated that extensive vascular networks could be generated in a co-culture system in a nanofibrous environment.

## 2. Materials and Methods

### 2.1. Preparation of Electrospun Fibrous Scaffolds

PCL/gelatin nanofibrous scaffolds were fabricated by using a previous electrospinning process [27]. The solutions for electrospinning were prepared by dissolving PCL (MW: 80,000; Sigma Aldrich, St. Louis, MO, USA) and gelatin (from bovine skin, Type B; Sigma Aldrich, St. Louis, MO, USA) (1:1 weight ratio) in 2, 2, 2-trifluoroethanol (TFE; Sigma Aldrich, St. Louis, MO, USA) to form a 10% *w*/*v* solution. A 7.5 kV positive voltage was applied to the PCL/gelatin solution via a 25-gauge stainless steel needle with a continuous flow rate of 1.0 mL/h using a syringe pump (NanoNC, Seoul, Korea) for 20 min at room temperature to generate randomly-oriented, electrospun PCL/gelatin nanofibers. The distance between the tip of the needle and the collecting plate was always at 15 cm. To make nanofibrous scaffolds with same size, cover glasses (18 × 18 mm) wrapped with clean aluminum foil were deposited on the collecting plate during the electrospinning process. 

The resultant fibers were crosslinked using a conventional vapor crosslinking method. Briefly, the PCL/gelatin nanofiber sheets were placed in a sealed desiccator containing an aqueous genipin solution (25 mg/mL in dimethyl sulfoxide) at room temperature for 24 h. The PCL/gelatin nanofiber mats were treated in a vacuum oven at 37 °C overnight to eliminate residual organic solvent from the electrospinning process and genipin followed by washing with Dulbecco’s phosphate buffered saline (DPBS). To confirm the crosslinking of gelatin, uncrosslinked and crosslinked PCL/gelatin nanofiber sheets were immersed in distilled water for 24 h and dried at room temperature for 12 h. The morphology of the resultant fibrous scaffolds was observed using scanning electron microscopy (SEM) with a model 7800 F apparatus (JEOL, Tokyo, Japan).

### 2.2. Isolation and Cultivation of hADSCs

We obtained human adipose tissues from the immediate transverse rectus abdominis musculocutaneous flaps of patients who underwent breast cancer surgery. We got agreements from patients to take adipose tissue at surgery and use them for research. All the experimental protocols using this patient-derived adipose tissue were approved by the Institutional Review Board (IRB, B-1612-374-305) for human subject protection at Seoul National University Bundang Hospital. The tissues were washed with phosphate buffer saline (PBS) containing 1% penicillin/streptomycin and minced with autoclaved scissors, followed by digestion with Dulbecco’s modified Eagle’s medium (DMEM; Welegene Inc., Daegu, Korea) containing 0.1% type I collagenase for 1 h at 37 °C. The tissues were filtered in the 50ml conical tube using a strainer and immersed in DMEM supplemented with 10% fetal bovine serum (FBS; CellSera, Rutherford, Austrailia) and 1% (*v*/*v*) penicillin/streptomycin. To obtain pure hADSCs, tissues were washed five times with saline and centrifuged at 1300 rpm for 5 min. Supernatant media and floating mature adipocytes were discarded. The pellets contained pure hADSCs. To determine whether the isolated cells were hADSCs, we observed the expression of the several surface makers of hADSCs using flow cytometry. Briefly, hADSCs at passage four were treated with trypsin– ethylenediaminetetraacetic acid (EDTA) and centrifuged, followed by washing with PBS twice. The collected hADSCs were filtered (35 μm nylon mesh) and diluted with 100 μL PBS in a facs tube (Corning Inc., New York, NY, USA). Each of the tubes was labeled as control (Mouse IgG), negative (CD19, CD45), and positive (CD44, CD73, CD90, CD105) using phycoerythrin-conjugated antibodies (BD Biosciences, San Jose, CA, USA) and wrapped using aluminum foil followed by incubation for 4 h with shaking at room temperature. Labeled hADSCs were analyzed using a flow cytometry device (BD FACS Aria II flow cytometer, BD Bioscience, San Jose, CA, USA).

The hADSCs were seeded in 75 T flasks and cultured in DMEM containing 10% FBS (CellSera) and 1% (*v*/*v*) penicillin/streptomycin at 37 °C in a humid atmosphere containing 5% CO_2_. After a day, the non-adherent cells were discarded, and the adherent cells were cultured in fresh medium. The medium was changed every two days. All the experimental protocols were approved by Seoul National University Bundang Hospital.

### 2.3. Cultivation of HUVECs

HUVECs (C2517A) were purchased from Lonza Inc. (Basel, Switzerland). The HUVECs were cultured on tissue culture flasks in endothelial basal medium (EBM-2, Lonza, Basel, Switzerland) with Single Quotes endothelial growth supplement (Lonza, Basel, Switzerland) and an antibiotic-antimycotic mixture (1% *v*/*v*) at 37 °C in a humid atmosphere containing 5% CO_2_. The medium was changed, and non-adherent cells were discarded every two days. When the cells reached 90~95% confluence in the culture flasks, HUVECs were subcultured to new flasks using 0.25% trypsin–EDTA solution. 

### 2.4. Co-Culture of ADSCs and HUVECs on Nanofibrous Scaffolds

To confirm the effect of the co-culture system on the vascular structure formation of HUVECs, electrospun PCL/gelatin nanofibrous scaffolds were utilized. First, the PCL/gelatin nanofiber scaffolds were immersed in the EGM-2 medium for 30 min to increase cell attachment at 37 °C. Next, ADSCs and HUVECs (2 × 10^5^ cells per scaffold, cell ratio of 1:3) were seeded on the nanofibrous scaffolds for co-culture system and monocultures were also established. In both the monoculture and co-culture systems, the total number of cells used for seeding was held constant regardless of the cells used. After allowing 2 h for the cell adhesion, the cells on the scaffolds were cultured in HUVEC growth medium for up to seven days. For the two-dimensional (2D) culture system, the same procedures were performed on a 24-well plate (SPL, Gyeonggi, Korea). To enhance the vascular structure formation of ECs and compare the effects of the growth factors on blood vessel development during 2D and 3D growth, S1P and VEGF were supplemented in the cell culture medium at concentrations of 250 nM and 50 ng/mL, respectively. These experiments were also carried using 24-well plates as a negative control. ADSCs and HUVECs were seeded onto the nanofibers and well plate at passage 4–6 and 5–7, respectively.

### 2.5. Cell Proliferation and Viability

#### 2.5.1. Cell Proliferation

To investigate the proliferation of ADSCs and HUVECs on scaffolds, a Cell Counting Kit-8 assay (CCK-8; Dojindo Laboratories, Kumamoto, Japan) was carried out at one, four, and seven days after cell seeding. Both cell types were seeded on the scaffolds (2 × 10^5^ cells per scaffold preparation for monoculture and co-culture groups) followed by the 10% *v*/*v* CCK-8 solution into the culture medium. After the samples were incubated for 4 h at 37 °C, cell proliferation was investigated by measuring the absorbance at 450 nm using a microplate reader (Biotek, Winooski, VT, USA).

#### 2.5.2. Cell Viability Assay

To assess the cell viability, a LIVE/DEAD^®^ Viability/Cytotoxicity Kit for mammalian cells (Invitrogen, Carlsbad, CA, USA), was used according to the manufacturer’s protocol. Briefly, both cells types were seeded on the scaffolds (2 × 10^5^ cells per scaffolds for both monoculture and co-culture groups). At seven days after culture, cell viability was measured by the exposure of cells to LIVE/DEAD solution (4 mM Calcein AM-green; live cells and 2 mM Ethidium homodimer-1-red; dead cells) for 30 min at room temperature. The cells were then visualized under a laser scanning microscope (Carl Zeiss, Oberkochen, Germany).

### 2.6. Immunofluorescence Analysis of Vascular Formation of HUVECs

For immunofluorescence staining, cell-laden scaffolds were washed and fixed in 4% paraformaldehyde for 10 min at room temperature, then permeabilized with 0.1% Triton X-100 in 1× PBS for 3 min at room temperature. After washing with 1× PBS, the cells were blocked using 1% bovine serum albumin (BSA) in 1× PBS for 1 h at room temperature before incubation overnight at 4 °C with primary antibodies against cluster of differentiation 31/platelet endothelial cell adhesion molecule (CD31/PECAM; 1:25 dilution; R&D Systems, Minneapolis, MN, USA) or podocalyxin (1:500 dilution; Abcam, Cambridge, UK). The samples were washed and then exposed to Alexa Fluor 568-conjugated secondary antibody (1:500 dilution; Abcam, Cambridge, UK) for 1 h at room temperature in the dark. F-actin was labeled with Alexa Fluor 488-conjugated phalloidin (1:300 dilution; Cell Signaling Technology, Danvers, MA, USA) and the nuclei were counterstained with 4′,6-diamidino-2-phenylindole, dihydrochloride (DAPI; Sigma Aldrich, St. Louis, MO, USA). To confirm the lumen structure formation, consecutive z-stack images of HUVECs labeled with phalloidin and DAPI in 2D, and 3D conditions, with an interval thickness of 3 μm, were acquired. The labeled cells were visualized using a laser scanning microscope. Cell length and aspect ratio values were obtained by analyzing the cell bodies expressing the junction proteins using the ZEN 2 software (Carl Zeiss, Oberkochen, Germany).

### 2.7. Statistical Analysis

All data are presented as the mean ± standard deviation. Each experiment was performed five times, and the differences of data were compared using one-way analysis of variance (ANOVA) followed by Tukey’s post hoc test. A *p*-value < 0.05 was considered statistically significant and is denoted as (*). The error bars represent the standard deviation between the experimental groups. Statistical analyses were conducted using the SigmaPlot 13.0 (Systat Software Inc., San Jose, CA, USA).

## 3. Results

### 3.1. Characterization of PCL/Gelatin Nanofibrous Scaffolds and hADSCs

**Figure 1:**
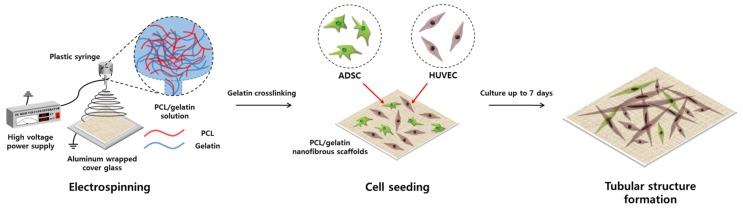
Schematic illustration of the PCL/gelatin nanofiber scaffolds using electrospinning process and co-culture system of ADSCs and HUVECs for blood vessel formation of the scaffolds.

**Figure 2:**
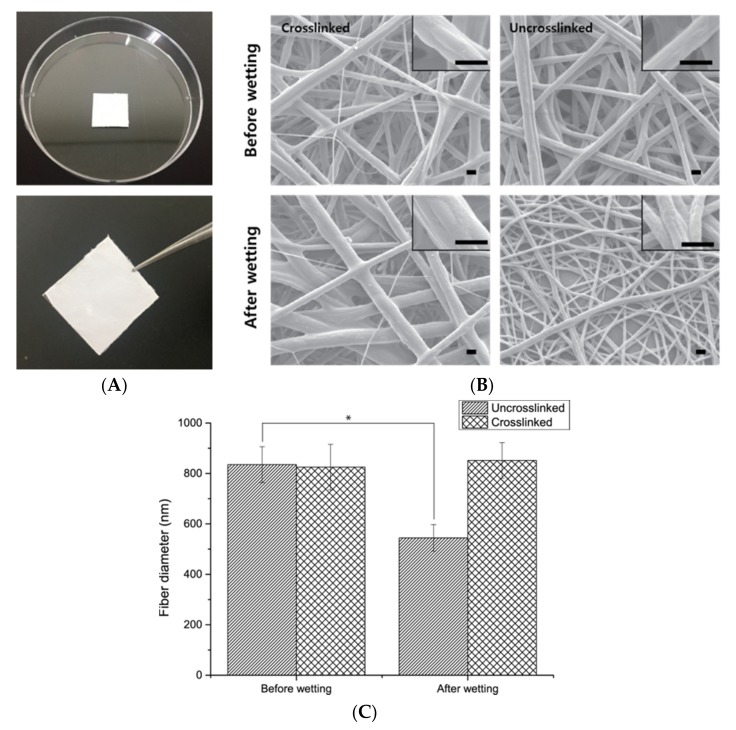
Characterization of the PCL/gelatin nanofibrous scaffolds. (**A**) Representative photographs of the scaffolds; (**B**) Representative SEM images of the scaffolds before and after wetting process. Scale bars 1 μm for low and high magnifications; (**C**) The diameter of the PCL/gelatin nanofibers before and after wetting process. One-way ANOVA; Tukey’s post hoc test; * compared to each group; * *p* < 0.001.

The electrospun PCL/gelatin nanofiber scaffolds were fabricated by the conventional electrospinning process described in Figure 1. Because PCL is a very hydrophobic polymer and gelatin has opposite properties, TFE was chosen as the solvent to dissolve both polymers. The resulting PCL/gelatin nanofibrous scaffolds had an approximate diameter of about 800 nm (835 ± 71.41 nm) and produced a 50 μm-thick matrix in a sheet form that was easily controllable using tweezers (Figure 2A). After the electrospinning process, the PCL/gelatin was treated with genipin under vacuum conditions to prevent the dissolution of gelatin in the aqueous cell culture medium during the experiment, since gelatin is a water-soluble material. The resultant crosslinked PCL/gelatin nanofiber scaffold had a similar diameter of fiber that was not crosslinked (825.2 ± 90.79 nm). The effectiveness of the genipin treatment for gelatin crosslinking was investigated by observing the different morphologies of the fibers before and after soaking in water. Before the wetting process, PCL/gelatin nanofiber scaffolds, irrespective of the crosslinking status, had a similar smooth surface structure, with no significant differences in morphologies between the two groups. This indicated that the genipin crosslinking process did not influence the fiber morphology. However, after the immersion of both types of nanofibers in water for 24 h, SEM examination revealed that the diameters of un-crosslinked fibers were significantly decreased (544.1 ± 53.18 nm) and the surface morphology of the fibers had become roughened, presumably due to the dissolution of gelatin in fibers during the wetting process (Figure 2B). The diameter of crosslinked fibers was slightly increased (851.3 ± 70.79 nm) after the wetting process, but not significantly different from the fiber diameter before wetting, with no evident surface change (Figure 2C). Therefore, the fabricated PCL/gelatin nanofiber consisted of bare PCL and successfully crosslinked gelatin. For the experiments, the cells (monoculture or co-culture) were seeded onto the crosslinked PCL/gelatin nanofiber scaffolds.

The flow cytometry was performed to characterize whether isolated cells are hADSCs. The cells were labeled with CD44, CD73, CD90, CD105 antibodies as positive markers and CD19, CD45 antibodies as negative markers. The labeled cells expressed positive markers of 98.69% for CD90, 94.78% for CD105, 86% for CD44, and 60% for CD73, respectively, while less than 0.2% for negative markers. The differentiation marker of hADSCs, CD73, was measured lower than other positive markers because this value varies greatly according to the patient’s age. However, the measured value of CD73 can represent that isolated cells maintain differentiation potency. This result indicates that the isolated cells are normal hADSCs (Figure S1).

### 3.2. Cell Proliferation and Viability

**Figure 3:**
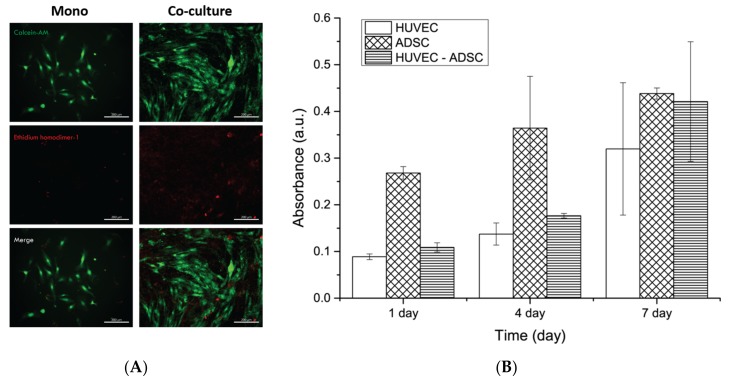
Viability and proliferation assays of monoculture and co-culture of HUVECs and ADSCs on the nanofibrous PCL/gelatin scaffolds. (**A**) Representative LIVE/DEAD fluorescence image of HUVECs and ADSCs in the co-culture system at seven days after culture. Scale bars represent 200 μm; (**B**) Cell proliferation assay of each group of cells (HUVECs only, ADSCs only, and co-cultured cells) cultured on PCL/gelatin nanofiber scaffolds. The CCK-8 assay was examined at one, four, and seven days of culture to identify the cell proliferation according to the absorbance at 450 nm.

The LIVE/DEAD assay was performed to determine the effects of the mono- and co-culture on the viability of HUVECs and ADSCs cultured on PCL/gelatin nanofiber scaffolds for seven days. In the LIVE/DEAD assay images, the green and red fluorescence indicated live and dead cells, respectively. Although a few dead cells were observed on the PCL/gelatin nanofiber scaffolds after seven days culture, no significant difference in cell death was evident among the monoculture and co-culture groups (Figure 3A). The CCK-8 assay was performed to determine cell proliferation on PCL/gelatin nanofiber scaffolds at one, four, and seven days after culture. HUVECs and ADSCs proliferated on PCL/gelatin nanofiber scaffolds in a time-dependent manner. The CCK-8 assay results indicate that cells in the monoculture groups and co-culture groups were able to proliferate on PCL/gelatin nanofiber scaffolds, which indicated that the PCL/gelatin nanofiber scaffolds were not cytotoxic and were able to maintain cell proliferation without meaningful cell death. (Figure 3B). 

### 3.3. Assembly of HUVECs in the 2D Culture System

**Figure 4:**
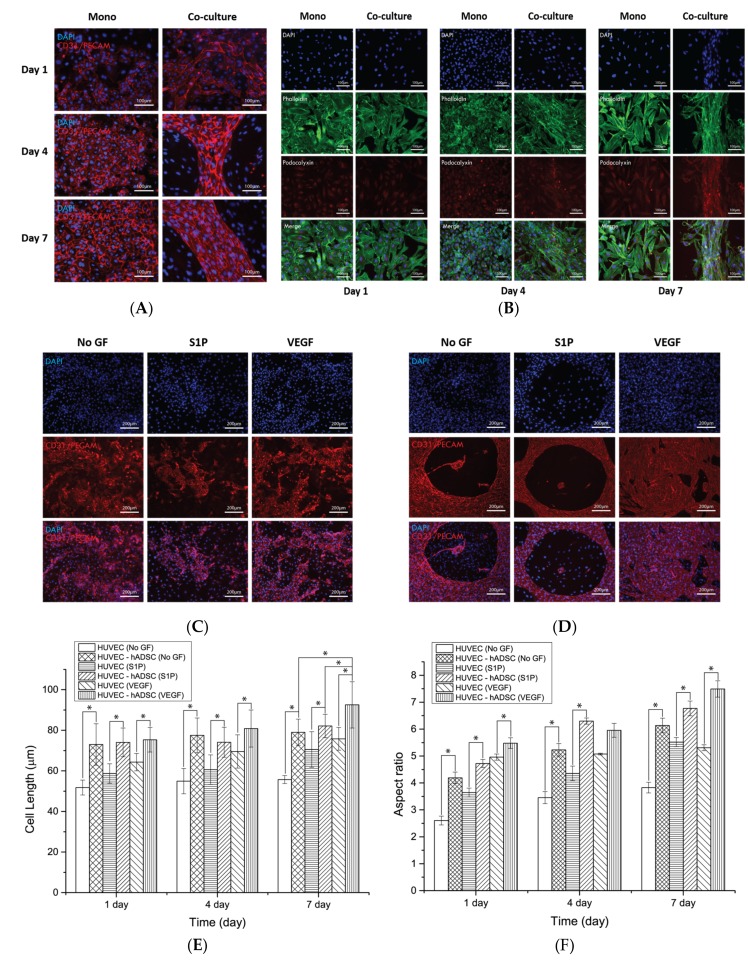
Blood vessel formation by HUVECs during mono- and co-culture growth in 24-well plates as assessed by immunofluorescence staining of blood vessel phenotypic markers. (**A**,**B**) Immunofluorescence imaging of the expression of endothelial cell markers: (**A**) intercellular junction protein CD31/PECAM and (**B**) podocalyxin at one, four, and seven days; (**C**,**D**) Representative immunofluorescent images of CD31/PECAM of HUVECs cultured in (**C**) monoculture system and (**D**) co-culture system to observe HUVEC behavior during different medium conditions in 24-well plates after seven days of culture; (**E**) Extent of HUVEC growth in 24-well plates under several culture conditions; (**F**) Aspect ratio of HUVECs in the 24-well plate under several culture conditions. One-way ANOVA, Tukey’s post hoc test; * compared to each group; * *p* < 0.001.

To determine whether the co-culture promoted vascular morphogenesis of HUVECs in the 2D culture condition, HUVECs and ADSCs were seeded in 24-well plates and cultured for one, four, and seven days, after which morphological changes and blood-vessel-like organization were compared with those of HUVECs alone cultured in other 24-well plates as a negative control. To observe the co-culture effect of ADSCs and HUVECs, we first investigated the expression of vascular endothelial tight junction molecules, CD31/PECAM, through immunofluorescence staining. As shown in Figure 4A, HUVECs cultured alone proliferated in a time-dependent manner and formed a cell monolayer with an increased number of tight junctions after four days culture with no differences in cell morphology. However, HUVECs cultured with ADSCs assembled to form blood-vessel-like structures, with the cells being significantly elongated. Next, the expression of another endothelial cell marker, podocalyxin, was investigated by immunofluorescence staining in all culture groups. In the monoculture group, podocalyxin was detected around the nuclei of HUVECs with a consistent degree of expression at all time points. In the co-culture group, the expression level of podocalyxin slightly increased after four days, and a morphological change into the assembled structure was observed through phalloidin staining (Figure 4B). Figure 4C,D depict the effect of growth factors on the blood-vessel-like structure formation of ECs in the monoculture and co-culture groups, respectively. As shown in the images, S1P and VEGF did not affect or enhance the formation of HUVEC monolayers or vascular morphogenesis in both groups, while HUVECs co-cultured in the presence of VEGF displayed significant differences in their cell length compared to other groups at seven days. The morphology of co-cultured HUVECs was significantly different in terms of cell length and aspect ratio compared to HUVECs cultured alone at all experimental days regardless of the presence or absence of growth factors (Figure 4E,F). These findings indicated that the growth factors did not affect vascular structure formation and that co-culture of ADSCs only promoted the assembly of HUVECs into the periphery of stem cells in the 2D culture system.

### 3.4. Blood-Vessel-Like Structure Formation of HUVECs on PCL/Gelatin Nanofiber Scaffolds

**Figure 5:**
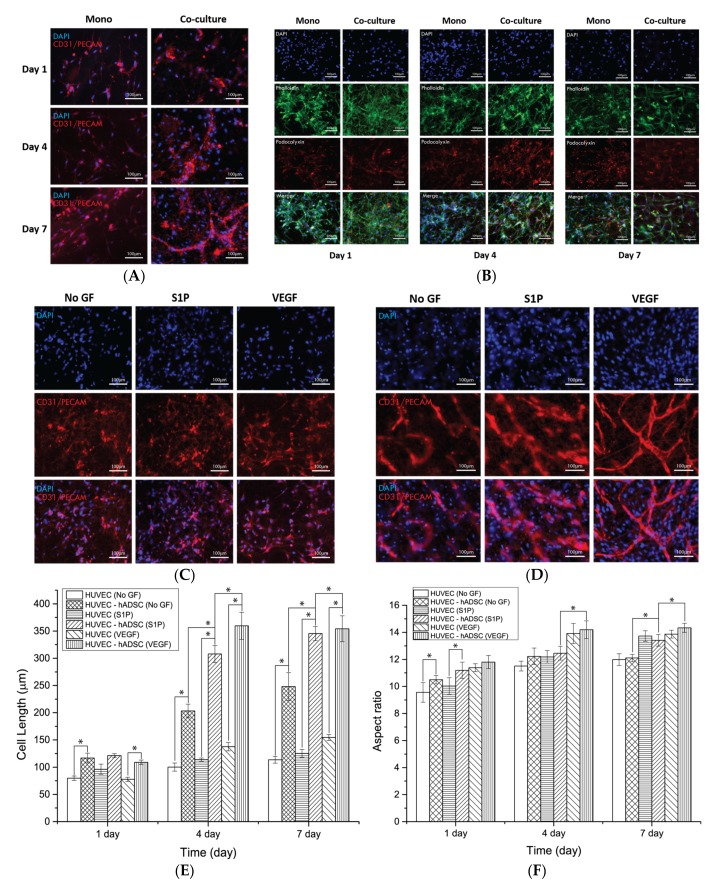
Blood vessel formation of HUVECs under mono- and co-culture on the PCL/gelatin nanofibrous scaffolds with immunofluorescence staining of blood vessel phenotypic markers. (**A**, **B**) Immunofluorescence images to express endothelial cells markers, (**A**) intercellular junction protein, CD31/PECAM and (**B**) podocalyxin at 1, 4, and 7 days; (**C**,**D**) Representative immunofluorescent images of CD31/PECAM of HUVECs cultured in (**C**) monoculture system and (**D**) co-culture system to observe HUVECs behavior under different medium conditions on the PCL/gelatin nanofibrous scaffolds after seven days culture; (**E**) Length of HUVECs on the PCL/gelatin nanofibrous scaffolds under several culture conditions; (**F**) Aspect ratio of HUVECs on the PCL/gelatin nanofibrous scaffolds under several culture conditions. One-way ANOVA; Tukey’s post hoc test; * compared to each group; * *p* < 0.001.

To further assess the vasculature morphology formation of HUVECs in the 3D culture system, the same experiments were performed on PCL/gelatin nanofiber scaffolds. Briefly, HUVECs were seeded on PCL/gelatin nanofiber scaffolds as mono- and co-cultures, and ADSCs were seeded as co-cultures, and then cells were cultured up to seven days. The expressions of the blood vessel phenotype markers, CD31/PECAM and podocalyxin, were evaluated through immunostaining. As shown in Figure 5A, monocultured HUVECs on PCL/gelatin nanofiber scaffolds proliferated in both the directions along the fibers, with significant differences in cell morphology between monoculture groups in the 2D and 3D culture environment. In addition, the level of expression of CD31/PECAM increased with cell proliferation and appeared throughout the cell membrane, unlike the 2D monoculture system. In case of the co-culture system, HUVECs also assembled with each other in peripheral ADSCs, but formed blood-vessel-like structures after seven days culture. Immunostaining of podocalyxin revealed no significant differences between the monoculture and co-culture groups in all culture periods (Figure 5B). However, when the growth factors were added to the culture medium, the behavior of the HUVECs was significantly different. Although the cell length and aspect ratio slightly increased with S1P and VEGF in the monoculture system (Figure 5C), the 3D culture system revealed a highly significant effect of the growth factors in the co-culture group for the formation of the blood-vessel-like network. The presence of growth factors promoted the formation of capillary-like structures and branch points (data not shown), and the inclusion of VEGF was associated with a more extensive blood vessel network than the other groups (Figure 5D). Quantitative cell length and aspect ratio measurements also revealed that the addition of VEGF was associated with more extensive cell assembly and elongation than other groups in both the monoculture and co-culture groups (Figure 5E,F). These results indicated that when cells are cultured in the nanofibrous environment, co-culturing with ADSCs and growth factors is effective in enhancing the blood-vessel-like structure formation of HUVECs.

### 3.5. Lumen Formation of HUVECs in the Monoculture Systems

**Figure 6:**
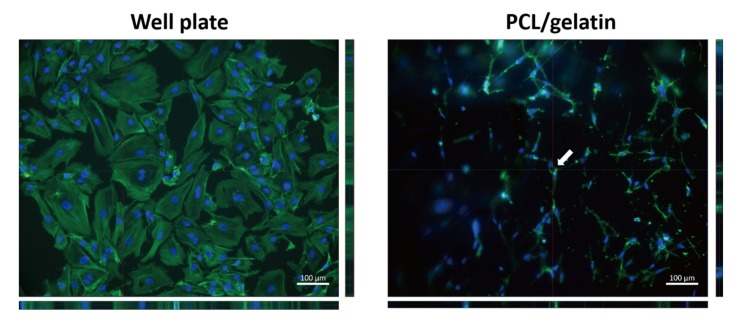
Lumen formation of monocultured HUVECs in the 24-well plate and the PCL/gelatin nanofiber scaffolds after seven days culture. The white arrow indicates the luminal structure of HUVECs. Scale bars represent 100 μm.

Next, we used laser scanning microscopy to assess the lumen formation from the ECs in the 2D and 3D culture conditions, which is an important aspect of vascular morphogenesis of ECs (Figure 6). Analysis of the reconstructed 3D configuration of the merged images of F-actin and DAPI revealed luminal structures in PCL/gelatin nanofiber scaffolds group at day seven. At day one, lumen formation was not evident with a uniform blood-vessel-like structure present (supporting information). However, hollow 3D lumens were unequivocally identified as being distributed on PCL/gelatin nanofiber scaffolds at day seven. No luminal structure was observed with HUVECs using the 24-well plate assay at day seven.

## 4. Discussion

In this study, we investigated the efficacy of the co-culture of HUVECs and ADSCs in vascular morphogenesis using PCL/gelatin nanofiber scaffolds, which were capable of cell adhesion, proliferation, and assembly into a blood-vessel-like structure. PCL/gelatin fibers were prepared from a uniform blend of PCL and gelatin. Bare PCL fiber is too hydrophobic for cell adherence and proliferation. However, the incorporation of the gelatin component of the ECM renders the fiber more hydrophilic and makes it biocompatible, both of which improve cell adhesion, proliferation, migration, and differentiation in the fibrous environment [28]. However, because gelatin can be easily dissolved in an aqueous cell culture environment, gelatin crosslinking is necessary to maintain the cell-friendly functions of PCL/gelatin nanofiber scaffolds. Although many studies used glutaraldehyde to crosslink gelatin to prevent dissolution during cell culture, glutaraldehyde is cytotoxic. Hence, we used genipin as the crosslinking agent. Genipin crosslinks spontaneously with proteins and some natural polymers [29]. On the contrary, because PCL fiber have mechanical properties that support cells in a manner reminiscent of natural ECM, incorporated PCL/gelatin nanofiber scaffolds can endure more vertical and lateral forces by cells than bare gelatin fibers. The fabricated PCL/gelatin nanofibrous scaffolds were biocompatible and allowed appropriate cell viability and proliferation regardless of the cell type during the cell culture periods (Figure 4A,B).

To observe the effects of co-culture and the presence of growth factors on blood vessel formation, HUVECs were cultured in a 2D system alone or along with ADSCs on PCL/gelatin nanofiber scaffolds with two different growth factors in the culture medium. The co-cultures were suspensions of ADSCs to HUVECs (1:3 ratio) that were seeded on the experimental surfaces. This ratio was used based on the prior finding that higher ADSC and HUVEC ratios can result in more abundant blood vessel regeneration on biocompatible scaffolds [30]. In these culture conditions, the two types of cells interacted through direct cell–cell interaction and a paracrine effect, in which soluble factors from other types of cells are shared. To enhance vasculature structure formation, we chose two different growth factors, S1P and VEGF, to supplement the culture medium. S1P is a pro-angiogenic factor that tightens the vascular-endothelial junctions and promotes vascular maturation through S1P signaling [31,32]. VEGF is commonly used for blood vessel regeneration because it induces the growth of pre-existing or new vessels in several organs [33]. Although a previous study observed that a cocktail of these growth factors enhanced the angiogenic sprouting of ECs, we chose to add the vasculogenic factors individually, as positive controls in the monoculture and co-culture systems. This was based on our hypothesis that cell–cell interactions between ADSCs and HUVECs are the main cue to enhance vascular structure formation.

Several recent studies reported that scaffolds from synthetic or natural polymers, such as gelatin [19], hydroxyapatite, calcium phosphate [20], and beta-tricalcium phosphate [30], have the potential to organize ECs into vascular phenotypic structures because cultured ECs could express junction proteins. Biomaterials with a coating of ECM proteins, fibronectin, or laminin have also been used to increase cell attachment or proliferation [34]. These biomaterial-based scaffolds provide a 3D cell culture environment and prevent cells from growing as a monolayer that leads to cell dysfunction. Among the various scaffolds, the nanofiber topography has many advantages over the conventional 2D culture system, because the nanofibrous environments mimic the architecture of the native ECM and promote cell adhesion, proliferation, and differentiation. In addition, the electrospinning process to generate nanofibrous scaffolds is quite fast compared to other methods, and the PCL/gelatin nanofiber scaffolds can create a cell-friendly and in-vivo-like environment without a protein-coating procedure. In this aspect, our co-culture system on PCL/gelatin nanofibrous scaffolds may be a promising and time-saving tool to regenerate blood vessels.

The morphological observations by the camera and SEM (Figure 2) indicated the successful crosslinking of gelatin in the fibers. Genipin is a natural crosslinker of proteins, collagen, gelatin, and chitosan; it is less cytotoxic than the conventional crosslinker, glutaraldehyde, or other synthetic crosslinkers. Gelatin is a natural polymer that is water-soluble and biocompatible for cells, and with relatively low mechanical properties compared with synthetic polymers. On the contrary, PCL is a Food-and-Drug-Administration-approved synthetic polymer used for implantation, drug delivery system, and tissue engineering, which features relatively low cell compatibility compared to the natural polymer. Having noticed that a mixture of PCL and gelatin in fiber form is effective for blood vessel regeneration, Jiang Y.C. et al. described the PCL/gelatin electrospun microfiber as having proper mechanical traits similar to natural human coronary arteries. However, they only studied the interaction between MSCs and PCL/gelatin fibers, not blood-vessel-like structure formation [35]. In this study, we confirmed the effect of blood-vessel-like structure formation using the PCL/gelatin nanofiber scaffolds and a co-culture system. Combining these properties, cells in monoculture or co-culture systems can attach and proliferate in the interconnected porous surface of the fabricated PCL/gelatin nanofiber scaffolds to a greater extent than on bare PCL fibers (data not shown). Cell proliferation and viability on the scaffolds were clearly observed (Figure 3). The F-actin staining results revealed the homogenous distribution of actin in the cytoplasm of ADSCs and HUVECs (Figure 5B). This result indicates that the crosslinked PCL/gelatin scaffolds successfully supported cell adhesion, proliferation, and spreading without pre-coating of proteins regardless of the cell type used.

Immunofluorescence examination of HUVECs in monoculture and co-culture systems on a 2D surface allowed the assessment of the blood-vessel-like structure formation. Monoculture of HUVECs in 24-well plates resulted in the formation of cell monolayers with a widespread formation of tight junction proteins similar to the typical findings with mammalian cell cultures on tissue culture plates. Surprisingly, HUVECs seeded with ADSCs assembled with each other to form cell-assembled monolayer structures over four days of culture (Figure 4). This may have reflected the direct cell–cell interactions between the two types of cells and the contribution of vasculogenic factors from the ADSCs, such as angiopoietin-1 and -2, VEGF, and interleukin-6, which induce the assembly of ECs similar to the de novo generation of blood vessels in vivo [36]. Additionally, because of the pericyte-like characteristics of perivascular ADSCs, cell compartmentalization between HUVECs and ADSCs was evident on the 2D surface, similar to the blood vessel morphology that consists of assembled ECs and peripheral pericytes (Figure 4A). However, HUVECs co-cultured with ADSCs in the 24-well plates did not show the capillary structure or cell sprouting. Furthermore, the addition of angiogenic factors to the mono- and co-cultures did not significantly change the behavior of HUVECs (Figure 4C,D). Only a slight increase in the proliferation, expression of junction proteins, and body length of HUVECs in VEGF-loaded co-culture group was observed. These results imply that cellular interactions in the co-culture condition mainly affect the behavior of HUVECs, rather than the angiogenic factors present in the 2D environment. The immunofluorescence results for podocalyxin shown in Figure 4B revealed the uniform distribution in HUVECs regardless of the culture condition. The expression was increased in a time-dependent manner in both the monoculture and co-culture groups. A previous study reported that podocalyxin-mediated control of vascular permeability and surface expression on the endothelial membrane precludes cell dysfunction and loss of blood flow [37]. These results support the present findings that HUVECs grown in mono- or co-culture on the 2D surface are functional, although blood-vessel-like structures do not form.

The immunofluorescence assay was also used to observe the expression of tight junction proteins and podocalyxin of HUVECs alone or with ADSCs on PCL/gelatin nanofiber scaffolds. The expression of the cell adhesion molecule CD31/PECAM-1 at the intercellular interface was also used to indicate the microvessel-like structure. In the monocultured HUVECs, the level of CD31/PECAM expression steadily increased and many tight junction proteins were observed on the scaffolds after 7 days of culture. Some elongated structure, but no apparent vasculature-like networks, were observed during the experimental periods. On the other hand, obvious microcapillary-like structure and blood-vessel-like networks were observed in the co-culture group. Co-cultured ECs with pluripotent stem cells or organotypic cells may facilitate the vascular network formation on 3D constructs since these types of cells can produce vasculogenic or angiogenic cytokines and factors [20,30,38]. Additionally, the ECM produced by ADSCs may promote the blood-vessel-like formation of ECs when these cells are co-cultured directly on the scaffolds. Although co-cultured HUVECs on the 2D surface also achieved the cell-assembled structure, there was no microcapillary-like structure. The latter emerged only on the PCL/gelatin nanofibrous scaffolds. This is probably the result of the provision of a natural ECM-mimicking environment by the PCL/gelatin nanofibers. In addition, angiogenic factors enhanced the microvessel structure formation of HUVECs in the co-culture groups (Figure 5D). In the co-culture system on our scaffolds, VEGF effectively triggered cell assembly, elongation, sprouting, and microvessel structure formation, whereas S1P was less effective than VEGF in the case of cell behavior. This is probably because VEGF is involved in all the vasculogenesis and angiogenesis processes of ECs. Recent studies compared the difference of cell behavior in the 2D and 3D environment. These studies identified that 3D cell culture models improved cell–cell interactions, cell–ECM interactions, protein expression, and gene expression than 2D cell culture models due to more in-vivo-like architecture, and our data also show that HUVECs in the nanofibrous environment more closely resemble the in vivo blood vessel networks than those in the 2D environment. This may support our results that growth factors work only in the nanofibrous environment because interactions between cells and growth factors more often occur in the 3D environment rather than the 2D environment. Furthermore, continuous expression of podocalyxin was observed in both the monoculture and co-culture groups and the elongation of HUVECs into blood-vessel-like structures was confirmed through the expression of protein at cell junction sites (Figure 5B). These results imply that the fabricated PCL/gelatin nanofiber scaffolds are effective for the formation of mature blood vessel networks.

To assess functional blood vessels, it is important to observe lumen formation, which is one of the key steps in vascular morphogenesis or new blood vessel development. In this study, the distinct lumen formation of HUVECs was observed only on PCL/gelatin nanofibrous scaffolds; no luminal structure was observed in 2D culture system at day seven of culture. Without any signaling molecules, HUVECs produced a lumen structure on the nanofiber topography. Our results demonstrated that functionally-engineered blood vessels could be successfully produced by culturing the cells on fabricated PCL/gelatin nanofiber scaffolds. Further studies will need to assess the cocktail effect of angiogenic factors, the cell distribution of luminal structure in the co-culture system on PCL/gelatin nanofiber scaffolds, and the anastomosis of the engineered new blood vessel with natural blood vessels.

## 5. Conclusions

The fabrication of PCL/gelatin nanofiber scaffolds using a conventional electrospinning process generated functional scaffolds, which provided a suitable environment for the new development of engineered blood vessels by HUVECs. These scaffolds consisted of a blend of hydrophobic PCL and hydrophilic gelatin. They were randomly oriented and showed proper biocompatibility. Co-culture of ADSCs and HUVECs on PCL/gelatin nanofiber scaffolds generated a mature blood-vessel-like network and increased the expression of tight junction proteins compared with monocultured HUVECs. The interaction with two angiogenic factors enhanced vasculature formation. The co-culture system of HUVECs and ADSCs on PCL/gelatin nanofiber scaffolds may be a promising approach for blood vessel regeneration. Furthermore, these in-vitro-engineered blood vessels could potentially be implanted into organ defects where tissue and blood vessel regeneration are needed.

## Supplementary Materials

The following are available online at http://www.mdpi.com/2079-4991/8/2/117/s1, Figure S1: Characterization of isolated hADSCs using flow cytometry. CD44, CD73, CD90, CD105 were labeled as positive markers and CD19, CD45 were labeled as negative markers.
