# Supplementary material for: Promotion of Vascular Morphogenesis of Endothelial Cells Co-Cultured with Human Adipose-Derived Mesenchymal Stem Cells Using Polycaprolactone/Gelatin Nanofibrous Scaffolds
Source: Nanomaterials (Basel). 2018 Feb 18;8(2):117. doi: 10.3390/nano8020117 (PMC5853748; doi:10.3390/nano8020117)
Supplement: Supplementary file 1 [file nanomaterials-08-00117-s001.pdf]

Promotion of Vascular Morphogenesis of Endothelial Cells Co-Cultured with Human Adipose-Derived Mesenchymal Stem Cells Using Polycaprolactone/Gelatin Nanofibrous Scaffolds

Yun-Min Kook, Hyerim Kim, Sujin Kim, Chan Yeong Heo, Min Hee Park, Kangwon Lee and Won-Gun Koh

Supplementary Figure

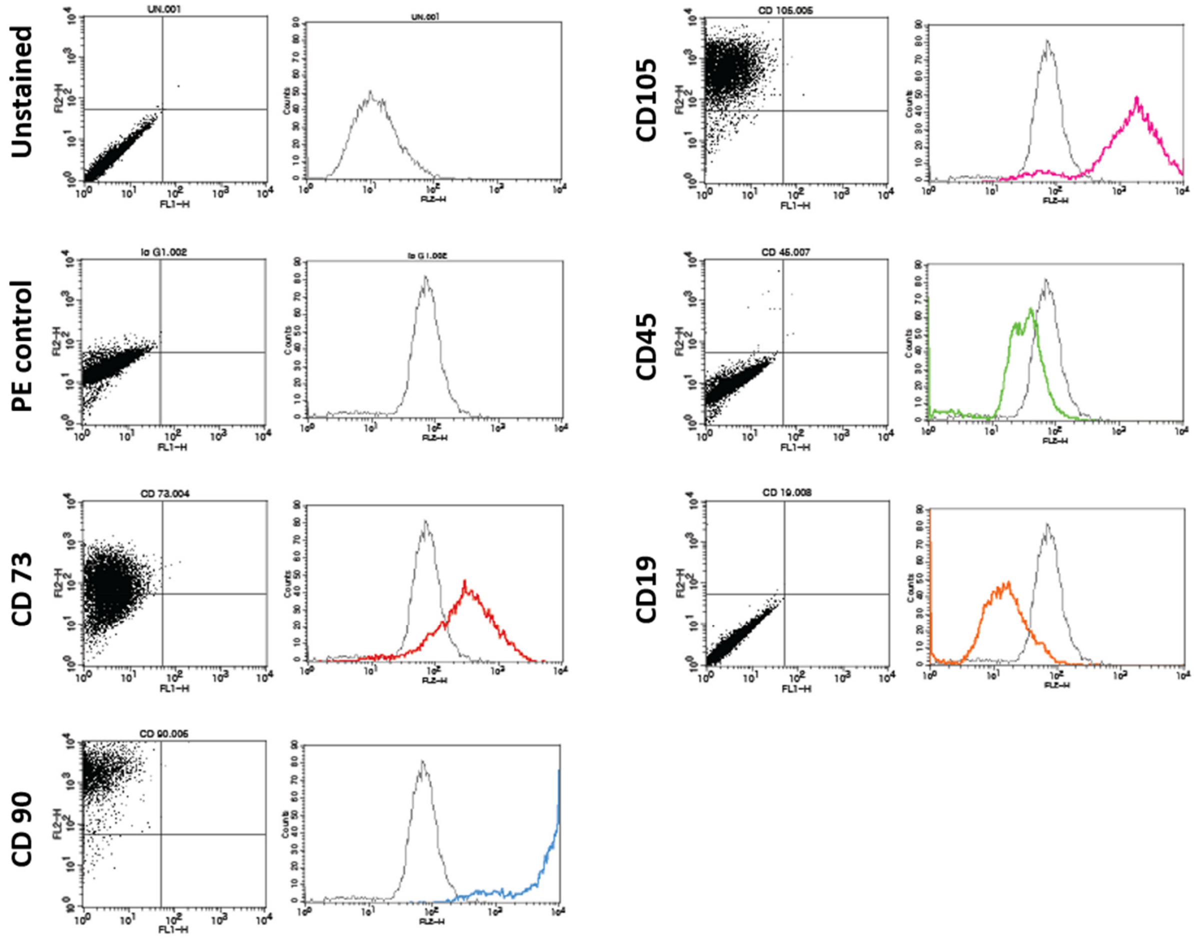

Figure S1. Characterization of isolated hADSCs using flow cytometry. CD44, CD73, CD90, CD105 were labeled as positive markers and CD19, CD45 were labeled as negative markers.
